# Multimodal artificial intelligence in urologic precision oncology: from algorithm to translational medicine (a systemized narrative review)

**DOI:** 10.3389/fonc.2026.1763359

**Published:** 2026-04-13

**Authors:** Farid Rajaee Rizi, Maryam Sadat Jamadi, Samin Rahimi, Moein Bighamian, Pouya Paidar, Mohammad Javad Taki, Nazila Bahmaie

**Affiliations:** 1 Endocrine and Metabolism Research Center, Isfahan University of Medical Sciences, Isfahan, Iran; 2 Department of Urology, Al-Zahra University-Affiliated Hospital, Isfahan University of Medical Sciences, Isfahan, Iran; 3 Department of Obstetrics and Gynecology, Faculty of Medicine, Isfahan University of Medical Sciences, Isfahan, Iran; 4 Department of Genetics, Faculty of Natural Sciences, Tabriz University, Tabriz, Iran; 5 Department of Urology, School of Medicine, Isfahan University of Medical Sciences, Isfahan, Iran; 6 Department of Computer Engineering, Faculty of Engineering, Graduate School of Natural and Applied Sciences, Gazi University, Ankara, Türkiye; 7 Department of Medical Physiology, School of Medicine, Isfahan University of Medical Sciences, Isfahan, Iran; 8 Department of Medical Biology, Faculty of Medicine, Ankara Yildirim Beyazit University (AYBU), Ankara, Türkiye

**Keywords:** artificial intelligence, genitourinary malignancies, molecular medicine, multimodal algorithms, precision uro-oncology, risk stratification, translation medicine

## Abstract

Precision oncology in urology increasingly depends on integrating heterogeneous data, including multiparametric imaging, histopathology, genomics, and clinical variables. Multimodal artificial intelligence (AI) offers a unified framework to manage this complexity, supporting refined risk stratification, personalized treatment decisions, and informed patient counseling. This narrative review examines applications of multimodal AI in prostate, bladder, and kidney cancers. Beyond listing individual tools, we emphasize how synergistic data fusion enhances the validation of diagnostic and prognostic performance. Clinical advances include more accurate tumor delineation on multiparametric MRI and predictive modeling of functional outcomes after surgery, underscoring the translational potential of these systems. However, major barriers hinder clinical adoption. Prospective validation remains scarce, data harmonization across institutions are limited, and the opaque nature of many algorithms fuels skepticism among clinicians. These factors collectively restrict the integration of multimodal AI into routine clinical practice. Closing this gap requires standardized data curation, development of interpretable and transparent models, and the design of collaborative human–AI workflows. Ultimately, successful translation will depend not only on technical progress but also on redefining trust and expertise in urologic oncology, ensuring that algorithmic insights are meaningfully aligned with bedside decision-making.

## 1 Introduction

As a highly prevalent and androgen-dependent malignancy of the male reproductive system, prostate cancer (PCa) ranges from indolent to aggressive, remaining as one of the most frequently-diagnosed cancers in men (1, 2). From an epidemiological perspective, PCa accounts for more than 1.4 million new cases annually, ranking as a major contributor to cancer-related morbidity and mortality worldwide, with the highest incidence of metastatic PCa observed in Australia/New Zealand, North America, and Western countries. Mortality rates are strongly dependent on predisposing genetic factors and tumor staging (Gleason score ≥ 3 + 4 for patients with clinically significant PCa) at the time of diagnosis (1–4).

Immunopathophysiologically, chronic inflammation, oxidative stress, and several cellular and molecular interactions between systemic inflammation and local immune response, as well as immune dysregulation, are critical determinants of malignant transformation within the tumor microenvironment (TME) of PCa (5). From a cellular perspective, PCa arises from the epithelial cells of the prostate gland, evading host immune surveillance by suppressing the functionalities of cytotoxic T lymphocytes, overexpressing immune checkpoint molecules (such as PD-1/PD-L1), and recruiting immunosuppressive cellular phenotypes (including regulatory T (T reg) cells and Myeloid-Derived Suppressor Cells (MDSCs)), worsening the prognosis in patients with PCa (6). In addition, altered secretion of cytokines and chemokines fosters a TME conducive to tumor progression, angiogenesis, and metastatic spread (particularly to the bone and lymphatic system, including locoregional lymph nodes) (6, 7).

Diagnostically and paraclinically, although serial measurement of serum prostate-specific antigen (PSA) levels is one of the most well-known sero-immune biomarkers, various types of prostate biopsies are also common diagnostic procedures for patients with PCa. These mainly include liquid biopsies (a clinically diagnostic and minimally invasive platform for isolating circulating tumor cells from blood samples of patients with PCa), digital rectal examinations (DREs), and surveillance (8–10). Diagnostically and clinically, despite the development of various types of computed tomography (CT) for PCa (specifically radiolabeled prostate-specific membrane antigen (PSMA) CT), positron emission tomography (PET)-based and magnetic resonance imaging (MRI)-based approaches have also been successfully considered as diagnostic procedures for investigating tumor initiation and progression in patients with PCa. These include PSMA PET, PSMA–PET/CT, fluorodeoxyglucose-PET/CT (FDG-PET/CT), and T2-weighted (T2w) images, apparent diffusion coefficient (ADC) images, diffusion-weighted magnetic resonance imaging (DWI), and dynamic contrast-enhanced (DCE) images (11–14). Although several successful results have been obtained from the aforementioned diagnostic procedures, mapping MRI-detected lesions to transrectal ultrasound (TRUS)-guided biopsies is still necessary (despite a 48% cancer detection sensitivity, as well as a high possibility for prostate deformation caused by rectal insertion of the TRUS probe). These issues make this approach time-consuming and challenging, potentially leading to missed diagnoses in patients with clinically significant PCa (15, 16). Moreover, paraclinically, PSA cannot be considered a specific sero-immune biomarker for patients with PCa because of a high positive predictive value for patients with benign prostatic hyperplasia (BPH) and prostatitis, as well as the existence of low or intermediate serum PSA levels in patients with clinically significant or metastatic PCa (17–19).

For higher-risk PCa, surgery [radical prostatectomy (RAPR)] and radiotherapy with androgen deprivation have been used therapeutically. Platinum-based chemotherapy (including cisplatin and carboplatin) has also been used clinically for small-cell or aggressive-variant PCa (20, 21). Androgen deprivation therapy (ADT) can also form the backbone of PCa treatment, most commonly through medical castration with gonadotropin-releasing hormone agonists or androgen receptor inhibitors (ARIs), including darolutamide and abiraterone. ADT can demonstrate acceptable effectiveness and improve median overall survival (OS) for patients with localized and metastatic hormone-sensitive PCa (mHSPCa) (2, 22–24). However, many studies report the frequent emergence of resistance to ADT, leading to metastatic castration-resistant prostate cancer (mCRPCa), which is associated with a poor prognosis (25). Recent advanced therapeutic modalities widely encompass next-generation ARIs, taxane-based chemotherapies (cabazitaxel or docetaxel), theranostics such as radioligand therapies (PET-compatible positron-emitting radionuclides for labeling radiopharmaceuticals, such as fluoride-18 [18F]), PSMA-targeted radioligand therapies (lutetium-177 PSMA therapy), molecular-targeted therapies, and immunotherapies (immune checkpoint inhibitors [pembrolizumab], new generation of therapeutic vaccines, and adoptive cell transfer) (2, 7, 26–28). Although these newly introduced approaches have the potential to extend survival in selected PCa patient populations, a durable remission remains elusive in patients with advanced PCa, leading to ongoing research toward precision oncology, molecular medicine, translation medicine, biomarker-guided therapies, omics-mediated therapies (proteomics, metabolomics, genomics, transcriptomics, and epigenomics), combination therapies, and artificial intelligence (AI) to enhance treatment efficacy and confer long-term clinical outcomes (24, 29–38).

Over the past decades, with substantial progress in AI—ranging from theoretical innovation to clinically applicable systems—it has rapidly emerged as a transformative tool in contemporary medicine (39), with profound implications for oncology, including urologic malignancies. Accordingly, it has been demonstrated that AI can assist urologists, oncologists, bioinformaticians, radiologists, cancer researchers, and basic medical scientists (40, 41) in radiologic interpretation, histopathologic analysis, automated pathology grading, diagnostic imaging, prognostication modeling, therapeutic decision-making, and therapy monitoring (39, 42–45). Nevertheless, the majority of these recently introduced AI models remain unimodal, relying solely on isolated datasets such as multiparametric MRI (mpMRI), digitized biopsy slides, or singular clinical variables (17, 46, 47). Although these unimodal AI systems have proven advantageous in clinical settings, reliance on single-modality inputs sharply contrasts with the inherently multimodal nature of clinical decision-making in uro-oncology, where radiologic findings, histological evaluations, integrated imaging, genomic or epigenomic pathways, molecular signatures, and patient-specific characteristics are collectively synthesized to inform individualized management strategies against urological malignancies (precision uro-oncology) (46–48). Moreover, this divergence between the unimodal orientation of current computational models like AI frameworks and the multidimensional nature of clinical decision-making for patients with PCa has created a critical translational bottleneck (49). Multimodal AI has been designed to assimilate heterogeneous data sources into a unified analytic model, capturing the intricate interplay among tumor biology, tumor heterogeneity, and patient variabilities. Such multimodal AI models offer a more realistic representation of tumor immunobiology in the TME of PCa, thereby holding promise for bridging the aforementioned gaps by enhancing clinical decision-making through improved risk stratification, optimized individualized therapies, and support for shared decision processes, ultimately redefining the future of uro-oncology precision medicine for patients with PCa (39, 50–52). Consequently, understanding the development, validation, and clinical translation of these multimodal AI algorithms has become essential for urologists, as these systems are intended to complement, rather than replace, clinical expertise (49, 53).

In this narrative review, the authors begin by outlining the conceptual underpinnings and technical frameworks of multimodal AI, including common strategies for data fusion architectures and recent methodological advances relevant to genitourinary malignancies (urologic oncology). They then examine the clinical applications of multimodal AI across diagnostic, prognostic, and therapeutic domains. In this section, they focus particularly on how these integrated models can improve diagnostic precision, optimize risk stratification, inform treatment planning, and predict post-operative or functional outcomes in patients with prostate, bladder, and kidney cancers. They also emphasize the role of transparency and explainability in clinician–AI interactions, approaches to mitigate existing biases, and strategies to ensure validity, reproducibility, and generalizability across diverse populations of patients with prostate or other urological malignancies. Subsequently, they outline a consolidated analysis of the practical considerations for embedding multimodal AI into multidisciplinary tumor boards of urologic oncology, identifying pathways for regulatory approval and quality assurance, and achieving routine patient-centered care. To that end, they propose a translational roadmap from algorithm development to clinical adoption. By consolidating current evidence and identifying future research priorities, they ultimately aim to synergize deep learning (DL) models and multimodal data to equip urologists, uropathologists, uro-surgeons, and uro-oncologists with both a conceptual framework and a critical lens, thereby implementing multimodal AI tools in real-world oncologic practice.

## 2 Methods

### 2.1 Study design

We conducted a systematic narrative review to describe the evolving landscape of multimodal AI applications in genitourinary diseases, with a particular emphasis on precision urologic oncology in patients with prostate, bladder, and kidney cancers across different clinical stages. This study was designed to reflect current developments in modern DL*,* multimodal data integration in oncology, and translational molecular medicine within modern oncologic practice.

### 2.2 Search method (search protocol)

To enhance the transparency and reproducibility of the literature selection process, the review methodology followed the reporting principles outlined in PRISMA, although the study itself was not intended to represent a formal systematic review or meta-analysis. The search strategy adhered to the academically accepted guidelines outlined in the “Preferred Reporting Items for Systematic review and Meta-Analysis Protocols (PRISMA-P)” statement from January 2020 (https://www.equator-network.org/reporting-guidelines/prisma-p/), (http://www.prisma-statement.org/), (https://www.prisma-statement.org/abstracts), as well as the PRISMA 2020 checklist—PRISMA statement. In addition, PRISMA 2020 statement checklists comprising a 27-item checklist for the manuscript and a 12-item checklist for the abstract were prepared.

### 2.3 Literature search strategy and screening process

A comprehensive electronic literature search was performed across multiple bibliographic databases, academic search engines, and digital libraries to identify relevant studies addressing multimodal AI in genitourinary malignancies, including COCHRANE Library, PubMed, Embase, MEDLINE, ScienceDirect, Scopus, System for Grey Literature in Europe (SIGLE) (to identify any theses and unpublished reports), Google Scholar, and Web of Science (WOS).

As a time interval, the search covered publications from January 2015 to June 2025, reflecting the recent expansion of DL and multimodal analytical frameworks in oncology. Search queries were developed using combinations of Medical Subject Headings (MeSH) (including Artificial Intelligence, Genitourinary Malignancies, Multimodal Algorithms, Molecular Medicine, Precision Uro-oncology, Translation Medicine) as well as their corresponding synonyms and related terminologies, and free-text keywords related to the core concepts of the review. Boolean operators (“AND,” “OR”) were applied to optimize search sensitivity and retrieval.

### 2.4 Eligibility (inclusion and exclusion) criteria

Eligible studies included a wide range of scientifically-published content on AI-driven or multimodal analytical approaches in genitourinary oncology. Included publications encompassed experimental and non-experimental (hypothesis) original research articles; review articles (including mini-reviews, best-evidence reviews, narrative (traditional) reviews, critical reviews, scoping reviews, transformative reviews, literature reviews, systematic reviews, and systematic review and meta-analyses); and comparative, cross-sectional, cohort, multi-center cohort, retrospective, prospective, viewpoint, observational, commentary, letter to the editor, editorial, opinion, short/rapid/brief communication, randomized clinical trial (RCT), case report, qualitative, quantitative, and case series articles, as well as matched-case control, and pilot studies. Conference proceedings, full-text/full-length articles, abstracts, dissertations, theses, book chapters, sections of books, and other gray literature sources were also considered to capture emerging evidence and findings.

Only studies available in English (or those providing an abstract in English) were considered. No hierarchical prioritization was assigned to specific study designs; rather, all eligible publications meeting the inclusion criteria were considered equally during the screening process.

### 2.5 Study selection and screening

The identification and screening of relevant publications were performed independently by six reviewers. Titles and abstracts were initially screened to determine relevance to multimodal AI applications in genitourinary diseases and precision uro-oncology. Potentially relevant articles were then evaluated through full-text assessment. Any discrepancies or disagreements during the screening process were resolved through discussion, and unresolved issues were referred to the corresponding author for final determination.

### 2.6 Manual search and reference screening

In addition to the electronic database search, a manual (non-electronic) backward reference search was conducted by reviewing the bibliographies of all included articles to identify additional relevant studies that may not have been captured in the initial database search.

### 2.7 Exclusion criteria

Studies were excluded if they contained irrelevant, insufficient, or ambiguous data; lacked clearly defined methodological descriptions; or did not adequately address the clinical or translational relevance of multimodal AI approaches in genitourinary disorders or precision urologic oncology.

### 2.8 Data synthesis

Given the heterogeneous nature of study designs and methodologies identified across the literature, findings were synthesized using a qualitative narrative approach, focusing on thematic integration of multimodal AI applications in diagnostics, prognostic modeling, molecular profiling, and precision therapeutic strategies in genitourinary oncology.

## 3 Results

### 3.1 Concepts of multimodal AI in precision urologic oncology

**Figure 1:**
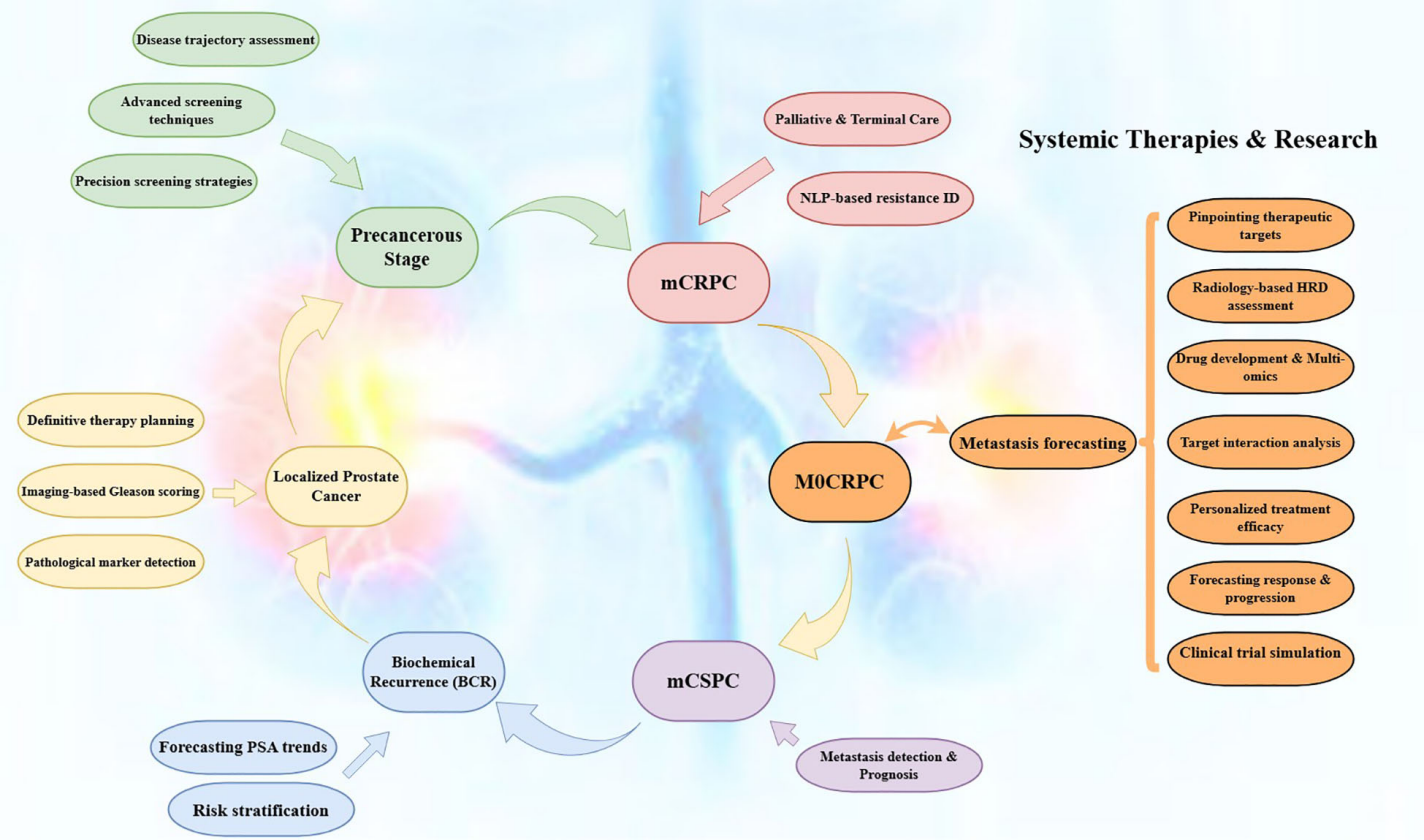
A schematic presentation of multimodal AI concepts in precision urologic oncology.

Multimodal AI in urological oncology represents a shift toward computationally synthesizing heterogeneous, high-dimensional datasets derived from clinical parameters with radiological, pathological, laboratory, and molecular data frameworks to enhance diagnostic accuracy, prognostic precision, and individualized treatment planning. This approach allows for modeling tumor biology, analyzing disease evolution or therapeutic outcomes, and advancing the clinical implementation of precision urologic oncology (**Figure 1**). Unlike unimodal approaches, multimodal AI leverages cross-domain feature interactions, enabling a more faithful representation of the biological and clinical complexity of urological malignancies, including prostate, bladder, renal, testicular, and upper tract urothelial cancers (54–56).

From a clinical data integration standpoint, multimodal AI incorporates structured and longitudinal variables such as demographic characteristics, comorbidity indices, symptom burden, treatment exposure, and temporal biomarker kinetics. Advanced machine learning architectures (e.g., ensemble models, attention-based Neural Networks (NN)) capture non-linear dependencies between patient-specific factors and disease trajectory, improving risk stratification, treatment selection, as well as prediction of clinical endpoints beyond traditional nomograms and regression-based tools (57, 58).

From the radiological *perspective*, multimodal AI integrates multiparametric MRI, contrast-enhanced CT, PSMA-PET/CT, and ultrasound datasets with clinical metadata. Radiomic and DL-derived features, encompassing tumor morphology, spatial heterogeneity, diffusion restriction, perfusion dynamics, and metabolic activity, are jointly analyzed to enhance tumor detection, local and systemic staging, and assessment of tumor aggressiveness. In PCa and bladder cancers, this integration improves discrimination of clinically significant disease, prediction of extracapsular extension, lymph node involvement, and early metastatic dissemination (15, 58–60).

From the pathological and histopathological domain, multimodal AI combines digitized Whole-Slide Images (WSI) with clinical imaging and molecular inputs. Convolutional Neural Networks (CNNs) extract quantitative morphometric and spatial features related to glandular architecture, nuclear pleomorphism, mitotic index, immune cell infiltration, and tumor–stroma crosstalk. When contextualized within multimodal models, these features enable more reproducible grading, refined prognostic classification, and identification of histologically occult high-risk phenotypes (61, 62).

From the laboratory and biofluid biomarker perspective, multimodal AI integrates serum analytes, urinary biomarkers, and inflammatory indices with imaging and pathology-derived features. Rather than relying on single-marker thresholds, AI models evaluate dynamic biomarker trajectories and multivariate interactions to distinguish indolent from biologically-aggressive disease, predict biochemical recurrence, and monitor Minimal Residual Disease (MRD) with improved sensitivity and specificity (63, 64).

From the molecular and multi-omics standpoint, multimodal AI incorporates genomic alterations, epigenetic signatures, transcriptomic profiles, proteomic patterns, and metabolomic landscapes alongside clinicopathological data. This integrative approach enables identification of molecular subtypes, oncogenic signaling pathways, and immune microenvironment characteristics associated with prognosis and therapeutic response. In urological cancers, such models inform precision medicine strategies, including selection for targeted therapy, immunotherapy, and combination regimens, while anticipating mechanisms of resistance (65, 66).

From a prognostic and predictive modeling perspective, multimodal AI enables time-dependent outcome modeling by assimilating longitudinal imaging, biomarker evolution, treatment interventions, and molecular changes. Survival analysis augmented by deep learning improves prediction of progression-free survival, cancer-specific survival, and overall survival, facilitating personalized surveillance protocols and adaptive therapeutic decision-making (67, 68).

### 3.2 Technical foundations of multimodal AI

Applying AI to discrete domains of urologic oncology to create a unified diagnostic model—such as CNNs trained to detect clinically significant PCa on mpMRI [like prostate imaging reporting and data system (PI-RADS) with 100% sensitivity], DL algorithms for automated Gleason scoring on digitized biopsy slides, and molecular biomarkers and genomic classifiers estimating recurrence risk—has demonstrated strong performance for highly sensitive detection and pixel-level localization of PCa (15, 17, 69–71). Although the advantages of unimodal frameworks should not be underestimated, these tools often underperform when externally validated on datasets, reflecting variability in imaging protocols, patient demographics, and healthcare delivery systems. They often fail to fully capture the multifaceted biological nature and clinical complexity of genitourinary malignancies, where the integration of imaging, histopathology, molecular profiles, and clinical data is critical for accurate diagnosis, efficacious treatment, and treatment planning monitoring (15, 61, 72). This has led to a translational bottleneck in which algorithms show promise in retrospective analyses but lack consistent real-world clinical applicability. These shortcomings underscore the growing momentum toward multimodal AI approaches, which seek to unify diverse data modalities into interpretable models better aligned with clinical workflows in urologic oncology (73).

Multimodal AI refers to computational frameworks capable of analyzing and integrating two or more of these heterogeneous data types, to improve predictive accuracy and clinical utility beyond what unimodal models can achieve (67, 74). In urologic oncology, these inherently heterogeneous data may include radiologic images (mpMRI, CT, and ultrasound, providing spatial and functional information about PCa morphology and heterogeneity), digitized histopathology (digitized WSI), enabling automated Gleason grading, PC subtyping, and TME analysis), genomic and transcriptomic profiles (including gene expression profiles, mutational analyses, and epigenetic markers, offering insights into PCa biology and aggressiveness), laboratory parameters, and clinical variables (patient age, PSA kinetics, disease comorbidities, predisposition disorders, and prior treatments, providing essential context for personalized risk stratification) (75–77).

### 3.3 Data fusion strategies

Integrating multimodal data poses significant methodological challenges, leading to three main fusion strategies: early, late, and hybrid fusion. Early fusion refers to the combination of raw or preprocessed data from different modalities prior to model training (at the input level before extraction). This requires careful data alignment and normalization but enables the model to learn joint representations from the outset. In late fusion, each unimodal model is processed independently, and the required features are also extracted independently. Afterward, their outputs are combined at a later stage (e.g., decision level) using ensemble methods or meta-learners. This modular approach can be advantageous due to its flexibility; however, the potential for missing complex inter-modal relationships should not be underestimated. Hybrid fusion combines elements of both early and late fusion by integrating intermediate representations, often through attention mechanisms or graph-based networks, to capture rich cross-modal interactions (78). These architectures aim to optimize predictive performance while maintaining model interpretability, facilitating the modeling of complex interdependencies across heterogeneous datasets.

Despite rapid advances in multimodal AI within radiology and oncology more broadly, comprehensive evaluations tailored to urologic oncology are still lacking. Existing literature tends to emphasize algorithmic development or focuses narrowly on individual tumor types, without providing a holistic view of how multimodal models could be deployed across prostate, bladder, and kidney cancers (73, 79). Additionally, practical issues central to clinical translation, including harmonization of heterogeneous datasets, algorithm interpretability, robust external validation, regulatory compliance, and incorporation into multidisciplinary tumor boards, are often addressed in a fragmented manner. This fragmentation has left a conceptual gap between technical innovation and clinical implementation, hindering clinicians’ ability to interpret predictions and understand the relative contribution of each modality. A dedicated synthesis that bridges these domains is therefore necessary to inform urologists and uro-oncologists on both the opportunities and limitations of multimodal AI (40, 80, 81).

Sometimes, in order to capture reliable clinical images for patients with PCa and prostate mapping in brachytherapy techniques, real-time TRUS and proper segmentation using MRI and TRUS-based data are essential. Accordingly, incorporating temporal information from TRUS-based images, an automatic prostate segmentation technique can be helpful in qualifying segmentation accuracy, as well as providing accredited TRUS-guided biopsies. This method proposes using deep CNNs for the registration of T2-weighted MRI and 3D TRUS volumes of the prostate, followed by a hybrid 3D/2D U-Net CNN, to increase the performance of PCa invasiveness assessment, prostate segmentation, and volumetric evaluation (82).

### 3.4 Representative architecture

Machine learning (ML), as a domain of AI, is a crucial technology for achieving AI. It extracts information from existing databases and generates intelligent interpretations, enabling integrative frameworks to simplify complex interactions among all clinical, pathological, physiological, biochemical, histological, biological, immunological, molecular, cellular, and genetic aspects of cancer (83).

DL architectures, as a subfield of ML, focus on deep artificial neural networks (ANNs), commonly including CNNs for imaging data (using a transfer learning approach), transformers for sequential or textual data, deep neural networks (DNNs), as well as graph neural networks (GNNs). They all have the potential to model the relationships between heterogeneous features, and their precise utilization in uro-oncology is no exception (17, 56, 84, 85).

Recent advances leverage attention mechanisms to focus on the most informative features across modalities, thereby enhancing interpretability and improving diagnostic and prognostic performance in patients with PCa as well as renal cell carcinoma (RCC) (86–88).

In this case, in a DNN-based algorithm aimed at automatic segmentation of the prostate gland in patients with PCa, the Dice similarity coefficient, Hausdorff distances, regional contour distances, and center-of-mass distances were evaluated, demonstrating a confirmed accuracy for the prognostic and diagnostic aims of the study (89).

To design more accurate and integrated computer-aided detection (CAD) tools, the use of an axial diffusion-weighted magnetic resonance imaging (DWI) procedure as a CNN architecture has been highly recommended to clinically differentiate patients with clinically-significant PCa (meaning to improve classifiers at the slice-level and patient-level PCa diagnosis by three dimensional (3D) CNN to feed DWI) (69). Other CNN architectures, such as DenseNet121, MobileNetV2, and EfficientNetB0, have also demonstrated the great potential of DL in increasing the accuracy and precision of medical image classification for patients with PCa (90).

### 3.5 Clinical applications of AI in precision urologic oncology

**Figure 2:**
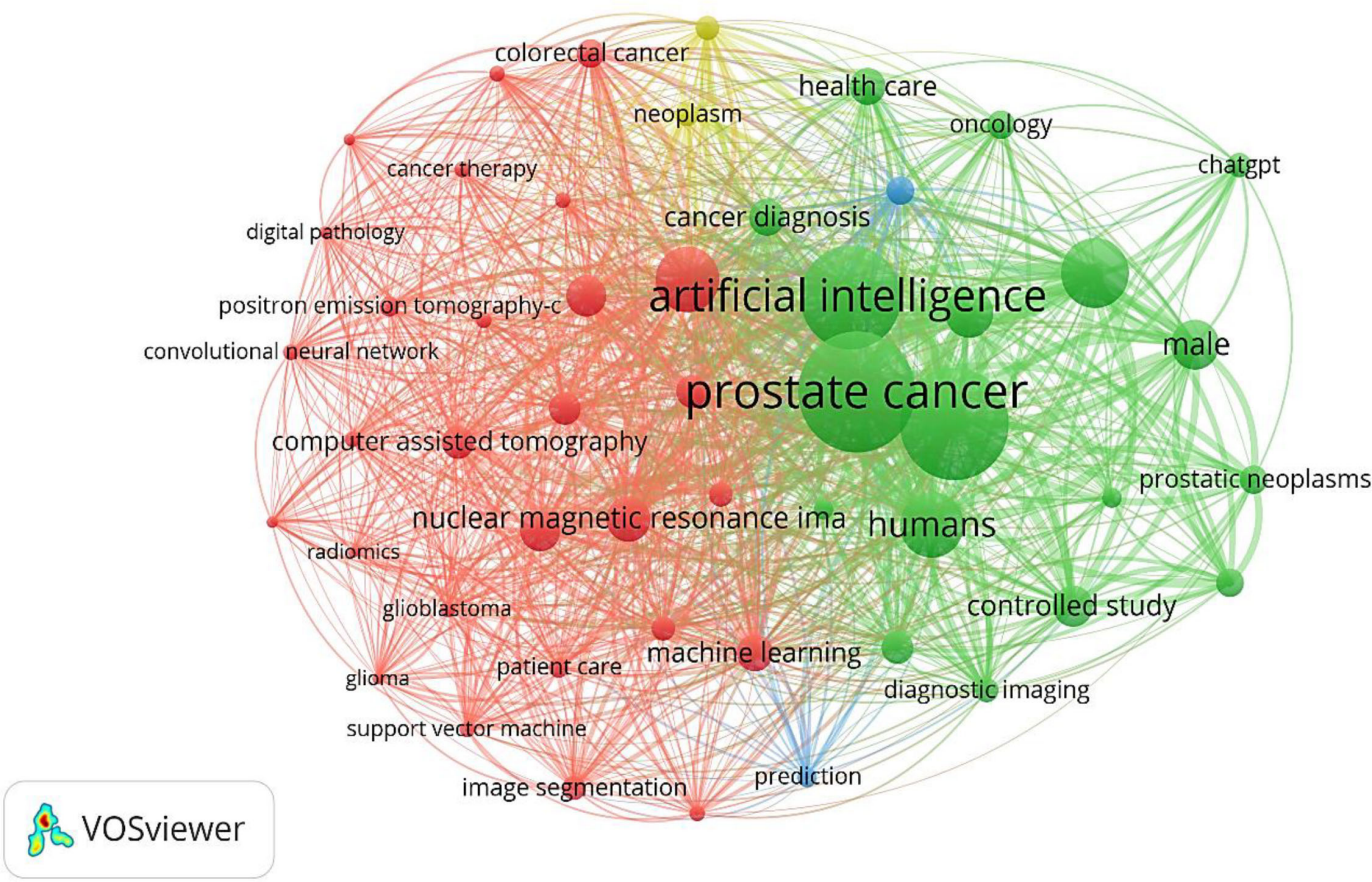
Clinical applications and emerging AI trends in PCa care.

Multimodal AI has demonstrated utility across diagnostics, prognostication, treatment planning, and functional outcome prediction in urologic oncology. Given the tedious nature of manual examination of nuclear segmentation and the large intraclass variations in histopathology images under high-resolution microscopes, there is a clear need for the development of AI-based techniques in diagnostic uro-oncology, as well as the integration of AI into radiomic models, using statistical and ML methods. Therefore, contouring radiation targets (specifying the irradiated segments), a critical step in prostate radiation oncology, can be automatically accelerated by AI (91–93). In prostate cancer, the integration of mpMRI-derived prostate cancer endotypes, histopathology, and genomic/epigenomic classifiers has significantly improved the detection of clinically-significant PCa, guided targeted biopsies, and refined active surveillance protocols. In bladder and renal malignancies, multimodal models like mpMRI and radiomics have been applied in cystoscopy, transurethral resection of bladder tumors (TURBT), multi-detector CT, and contrast-enhanced MRI to predict tumor staging and risk of recurrence, and monitor therapeutic responses, thereby enhancing precision in clinical decision-making (**Figure 2**) (94–98).

#### 3.5.1 Therapeutic values of AI in precision urologic oncology

Multimodal AI and multi-omics fusion analysis enable individualized treatment strategies. By integrating patient-specific molecular and clinical data with imaging and histopathology, these models can predict therapeutic efficacy, reduce overtreatment, and optimize surgical or systemic interventions. Furthermore, they facilitate forecasting of functional outcomes, such as urinary continence or renal function, supporting patient-centered care and informed shared decision-making (65, 94, 95).

#### 3.5.2 Predictive values of AI in precision urologic oncology

AI-based algorithms not only offer clinical benefits by increasing diagnostic accuracy, but have also demonstrated impressive value in predicting PCa metastasis and radiotherapy toxicity, using combined histopathology and clinical-pathologic parameters (WSIs of RAPR, core needle biopsy samples, or tissue microarrays) in surgically-treated patients who had undergone a RAPR (99–101).

Additionally, deep CNNs were used to develop AI-based models aimed at predicting the risk for early recurrence in patients with PCa, as well as novel identification of cancer driver regions (regardless of their Gleason grading), using visual and subvisual morphologic features from WSI (102). Furthermore, in an interdisciplinary study, a biologically informed DL model (named P-NET) was designed to stratify patients with PCa who had revealed treatment resistance, and evaluate the molecular drivers of treatment resistance (like *MDM4* and *FGFR1*), using a DNN-based algorithm (**Figure 2**) (103).

Here, it seems that development of a Prostate Cancer Risk (PRISK) tool using AI-based multimodality integration with radiomics ML and DL algorithms, as well as a Med3D DL network incorporating pre-operative PSMA-PET/CT imaging, can be of clinical significance in providing a precise risk assessment. This could consequently lower the overtreatment burden and inform clinical decisions by radio-oncologists or interventional radiologists regarding procedures like extended pelvic lymph node dissection (ePLND) for patients with clinically localized PCa, pelvic lymph node metastasis (PLNM), or pelvic lymph node invasion (PLNI) (104, 105).

In addition, given the challenges in predicting the timing of genomic changes during the transition from castration-sensitivity to castration-resistance in patients with metastatic castration-sensitive prostate cancer (mCSPCa) using pathology specimens, and the lack of less invasive, time-preserving approaches, it has been shown that ML-based analysis of cell free DNA can facilitate a precise predictive and prognostic approach. This can inform subsequent and optimized therapeutic procedures for these patients with PCa (106, 107).

#### 3.5.3 Diagnostic accuracy enhancement by AI in precision urologic oncology

Beyond therapeutic and predictive values, multimodal AI systems can diagnostically improve tumor detection, differentiation of malignant tissues from benign tissues, and tumor characterization by combining imaging data, histopathologic features, immunohistochemical staining (IHC), and clinical variables (**Figure 2**) (106, 108). For example, in PCa, models integrating mpMRI with biopsy histology and sero-immune biomarkers have demonstrated advanced imaging and superior accuracy in distinguishing clinically-significant disease from indolent lesions, compared to any unimodal approach alone. Such a precisely enhanced diagnostic tool may reduce adverse consequences, such as unnecessary biopsies and overtreatment in patients with PCa (58, 109–111).

To define involved lymph nodes and organs, and for pathologic staging of patients with PCa, an ANN model can predict the final pathologic stage of the disease by integrating pre-operative pathologic and clinical features (including pre-operative serum PSA level, clinical Tumor, Lymph node, and Metastasis (TNM) classification, and Gleason score from the biopsy specimen) (112).

#### 3.5.4 Risk stratification and prognostication by AI in precision urologic oncology

Accurately predicting tumor aggressiveness and the risk of tumor recurrence are considered determinant factors in precisely tailoring clinical outcomes for patients with PCa (113, 114). Multimodal AI models incorporating genomic signatures (large-scale cancer genomics datasets like the ReIMAGINE Multimodal Warehouse, or data with over 250 parameters per participant containing demographics, clinical baseline characteristics, bpMRI information, secondary care MRI, and biopsy information from the Movember GAP3 project), alongside radiomics data, imaging, and clinical factors, provide more robust stratification tools for these patients (**Figure 2**) (50, 115, 116).

More interestingly, accurate risk stratification in patients with localized PCa receiving neoadjuvant ADT and radiotherapy can be accelerated by predicting possible biochemical recurrence events (characterizing tumor aggressiveness, extracapsular extension, and seminal vesicle involvement) even 10 years after diagnosis or RAPR. This can be achieved by integrating putative prostate region-wise imaging biomarker (radiomic, diffusion, and/or perfusion features) profiles extracted from MRI images (117, 118).

Results from a wide range of studies highlight the role of assessing treatment response and predicting survival rate in patients with non-muscle invasive bladder cancer (119, 120). For instance, results from a wide range of multidisciplinary research on patients diagnosed with bladder urothelial carcinoma demonstrated the ability of multiparametric radiomic features with thin-slice enhanced CT, accompanied by other comprehensive clinical indicators, to pre-operatively predict pathological grade (low or high) in these patients (121, 122).

To evaluate the risk incidence of side-specific extra-prostatic extension (EPE) and subsequently select nerve-sparing RAPR, the development of ML through integrating pre-operative clinicopathological variables can meet the criteria to aid onco-surgeons in counseling patients with PCa and making informed and confirmed treatment modality selections (123).

Due to the underestimation of intra-prostatic extension (IPE) by MRI, validation of focal treatment margins should be considered for patients with PCa. The development of AI can simplify this by incorporating multimodal imaging and biopsy with a higher mean sensitivity for patients with an intermediate-risk of PCa who have undergone RAPR. Hence, AI is expected to reduce cancer recurrence and standardize treatment margin definition for patients with surgically removed prostate specimens, demonstrating a negative margin as a reliable feature for onco-surgeons to precisely counsel their patients with PCa (124). Accordingly, **Table 1** highlights the most novel studies using clinical applications of AI in precision genitourinary malignancies and uro-oncology.

**Table 1:** Clinical applications of AI in precision uro-oncology.

No.	Type of the study	Aims of the study	Patients	Algorithm/model	Authors (year)	Results	Limitations	Reference
1	A multicenter cohort study.	Exploring the value of using integrated multimodal information for International Society of Urological Pathology (ISUP) grading and prognostic stratification in clear cell RCC (ccRCC) patients, to guide post-operative adjuvant therapy.	512 ccRCC patients who underwent radical or partial nephrectomy at the hospital, 175 ccRCC patients from The Cancer Genome Atlas (TCGA) cohort, and 42 ccRCC patients from the Clinical Proteomic Tumor Analysis Consortium (CPTAC) cohort.	A multimodal predictive signature (MPS).	Zheng Qingyuan et al. (2025)	Demonstrating high accuracy in ISUP grading for patients with ccRCC, being used for precise detection of nuclear grading, assisting clinical decision-making.	Necessity to validate in the form of prospective research (according to the inherent biases and unknown confounding factors), as well as an imperative need to integrate features from multiple CT phases, in order not to miss any useful points.	(125)
2	A prospective, single-arm pilot single-center validation study.	Showing the applicability of the AI-enhanced simulated Raman histology (SRH) to samples taken directly from prostatectomy specimens, validating the AI model developed by Mannas et al., to assess margin samples obtained directly from the excised prostate gland.	Patients with PCa, undergoing robot-assisted RAPR, histologically confirmed prostate adenocarcinoma classified as intermediate or high risk according to the European Association of Urology classifications.	New York University AI algorithm (NYU-AI), based on the Inception-ResNet-v2 CNN architecture.	Arif Özkan et al. (2025)	Supporting the potentials of SRH, robustness and generalizability of the NYU-AI approach for intraoperative detection of positive surgical margins during RAPR, in a new anatomic context against conventional frozen-section and paraffin-embedded histology.	Existence of a small patient cohort, the single-center design, previous training of the NYU-AI tool on prostate biopsy and periprostatic surgical-bed samples, and the lack of testing of interobserver agreement.	(126)
3	A retrospective multi-center study.	Constructing a multimodal imaging DL model, integrating mpMRI and 18F-PSMA-PET/CT for the prediction of EPE in patients with PCa using the EPE-grade scoring system, assessing its effectiveness in enhancing the diagnostic accuracy of radiologists.	Pathologically confirmed PCa patients who underwent PARP.	A multimodal DL model incorporating mpMRI and 18F-PSMA-PET/CT.	Fei Yao et al. (2025)	Depicting promising predictive performance for EPE in patients with PCa, enhancing the accuracy of radiologists in EPE, holding potential as a supportive tool for more individualized therapeutic decision-making.	A limited dataset size despite existing retrospective analysis, a limited interpretability of the DL model, as well as a modest sample size for both model construction and external validation.	(127)
4	A retrospective study.	Validating a novel ML model to predict early progression (≤ 12 months) in patients with mCRPCa, comparing its performance against standard ML algorithms.	172 patients with mHSPCa.	A novel rivality index (RINH)-based model, adapted from chemo-informatics.	Miguel Ángel Gómez-Luque et al. (2025)	Offering a robust tool for risk stratification in patients with mHSPCa, being capable of personalizing therapeutic strategies.	A retrospective design and use of a single-institution data set.	(128)
5	Research article: diagnostic accuracy study	Evaluating the noninvasive predictive value of 18F-PSMA PET/CT and mpMRI in assessing for dual-parameter risk stratification in patients with PCa and Ki-67 expression.	84 patients with PCa, who underwent 18F-PSMA PET/CT and mpMRI before or after biopsy, as well as 11 patients with PCa, who underwent 18F-PSMA PET/CT and mpMRI examinations prior to or within 4 to 6 weeks following puncture biopsy.		Aihemaitijiang, Mierzhayiti et al. (2025)	Enhancing PCa risk assessment and predicting Ki-67 expression, indicating potential for disease progression and metastasis, through SUVmax/ADCmin, introducing it as a key imaging parameter for evaluating tumor biology, guiding treatment strategies.	Existence of a small sample size of patients with PCa, leading to a limited generalizability and statistical validity of the results, conducting the study at a single-center, leading toward bias and geographical constraints, as well as absence of gross specimens for pathologic grading and reliance solely on biopsy data in patients who had not undergone RAPR.	(129)
6	A retrospective cohort of patients-based study.	Exploring the prognostic significance of tumor burden quantification, derived from PSMA-11 PET/CT, using AI.	107 consecutive patients with mCRPCa, receiving Lutetium-177 (^177^Lu) PSMA therapy.	The algorithm is constructed based on the nnU-Net framework with the Residual Encoder Layer (ResEncL) architecture. The model was trained on a multi-tracer dataset comprising 475 FDG-PET/CT, 408 PSMA-PET/CT, and 410 DOTATATE-PET/CT studies (all independent of the current study’s validation cohort).	Shiming Zang et al. (2025)	Revealing prognostic significance of AI-based volumetric analysis of tumor burden on PSMA PET for survival in ^177^Lu-PSMA-treated mCRPCa patients, might help in personalized risk stratification, and facilitate AI-aided therapeutic decision-making.	Measuring out the study as a retrospective one, as well as a limited sample size.	(130)
7	Experimental study.	Investigating a novel multiphase classification framework, combining YOLOv8 for high-accuracy RCC grading and GradCAM for enhanced model interpretability.		A-publicly available KMC Kidney Histopathology Dataset.	Amna Bamaqa et al. (2025)	Not only providing superior diagnostic performance, but also offering transparent decision-making, being crucial for clinical adoption.	The reliance on a single dataset for training and evaluation, leading toward biases that limit the model’s ability to generalize.	(131)

### 3.6 Translational challenges in multimodal AI in precision genitourinary malignancies

**Figure 3:**
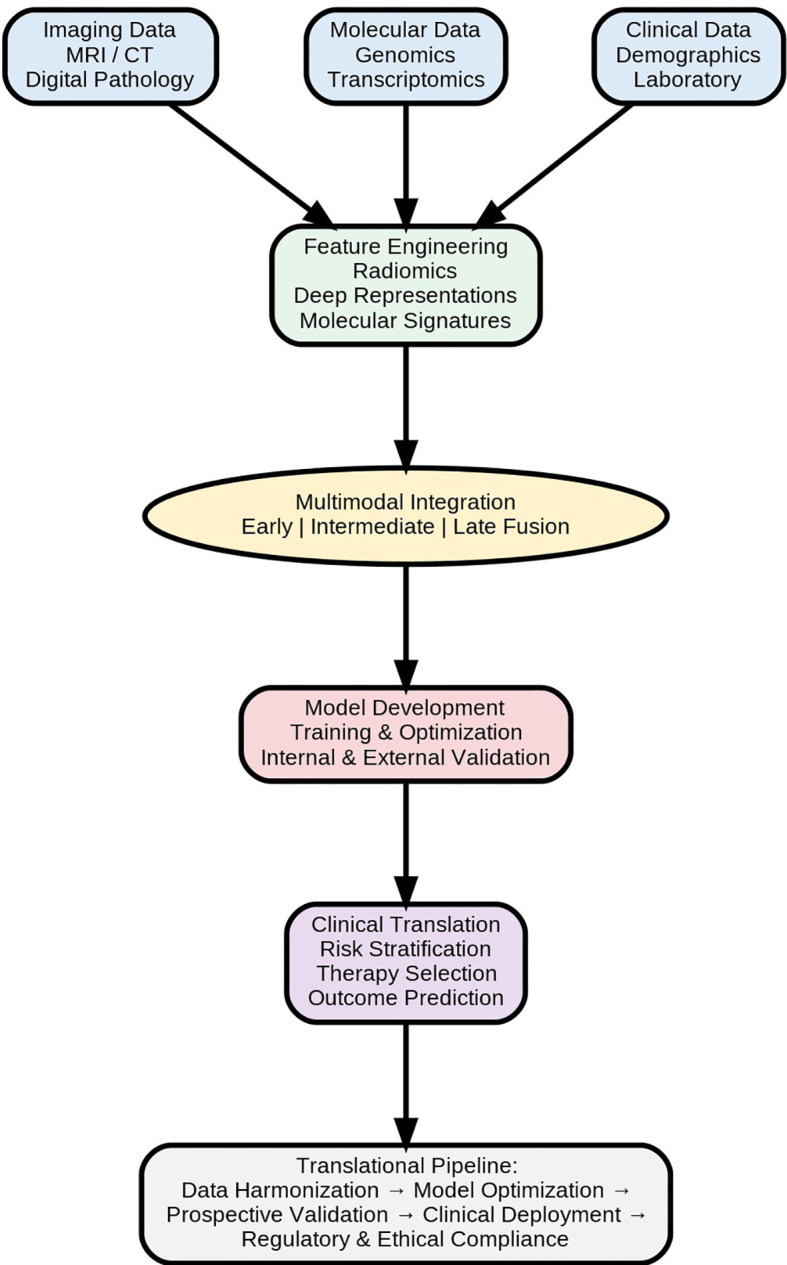
Multimodal AI in precision and translational genitourinary malignancies.

Despite their promise, several barriers impede the widespread clinical adoption of multimodal AI in precision genitourinary malignancies, generally including difficulties in translating multi-omics data into clinically-utilizable biomarkers to find a correlation between methodological rigor and clinical relevance for patients with PCa (**Figure 3**) (132). Furthermore, variability in data acquisition at different levels, significant dimensionality with a wide array of variates and covariates, difficulties in integrative analyses, differences in imaging protocols, variety in histopathology processing, reading mpMRI images with reduced quality, moderate inter-reader reproducibility of data acquired via mpMRI, and complicated laboratory assays compromise the model’s generalizability for users (132–134).

It is worth mentioning that bias in training datasets, as well as a lack of standardization and reproducibility in each step of mpMRI image processing (or even in classifier implementation) can result in inconsistent performance across demographic groups (the studied population of patients with PCa), highlighting the importance of external validation. For successful integration into clinical workflows, factors such as interpretability, clinician engagement, regulatory compliance, and ethical considerations should be taken into account (132, 133, 135).

Addressing these challenges necessitates a structured translational roadmap extending from algorithm development to clinical deployment. Key components include harmonization of multi-institutional datasets, prospective validation, incorporation of explainability mechanisms, and adherence to regulatory standards. Embedding multimodal AI within multidisciplinary PCa tumor boards, where radiologic, pathologic, histologic, genomic, epigenomic, immunologic, and clinical insights converge, is essential to ensure contextually relevant and actionable recommendations for clinical specialists (135).

### 3.7 Treatment planning and personalized therapy selection by AI in precision genitourinary malignancies

By synthesizing heterogeneous patient data, multimodal AI transformatively assists clinicians in refining risk stratification of patients with genitourinary malignancies, defining tumor volume, and selecting optimized treatment modalities, ranging from active surveillance (the standard for patients with a low risk of PCa development) and focal therapies (for patients with clinically significant PCa) to precise robotic-assisted RAPR and systemic therapies (for patients with localized PCa), with minimized adverse effects (136–139). It has been postulated that merging models can promisingly predict responses and clinical outcomes after administration of focal cryoablation therapy, neoadjuvant chemotherapy, or immunotherapy through integrating MRI-guided biopsies, baseline tumor characteristics, and genomic profiles. This could potentially guide personalized treatment pathways for these patients (136, 140–144).

Interestingly, by integrating complete records of clinicopathological variables and follow-up results, AI development can accurately create a platform for novel identification of patients with localized PCa who can benefit from surgery or radiotherapy modalities, offering radio-oncologists and surgeons an informed and confirmed choice for selecting the most efficient treatment modalities (145).

### 3.8 Prediction of functional outcomes by AI in precision genitourinary malignancies

Beyond oncologic control, clinical applications of AI in precision genitourinary malignancies include preserving post-operative functions like urinary continence, erectile function, and sexual function, which remain paramount in patients with PCa. It has been demonstrated that multimodal AI-based tools can forecast post-treatment complications, such as incontinence and erectile dysfunction, even at 1 to 12 months post-surgery, by analyzing pre-operative imaging, patient comorbidities, and surgical variables through machine-learning ANN in patients with localized PCa (146–148). These predictive insights support shared decision-making and patient counseling regarding expected quality-of-life (QOL) outcomes, with acceptable simplicity and interpretability for clinicians (146–150).

### 3.9 Enhancing multidisciplinary collaboration in precision genitourinary malignancies through AI use

By delivering explainable risk assessments and predictions of clinical outcomes (oncologic and post-operative), multimodal AI platforms facilitate communication within multidisciplinary tumor boards. The capacity to integrate complex data streams into coherent, clinically interpretable outputs enhances collaborative decision-making among oncologists, nephrologists, uro-surgeons, uro-oncologists, interventional radiologists, radiation oncologists, pathologists, clinical researchers, bioinformaticians, computational experts, cancer biologists, technology providers, ethicists, health policy makers, and healthcare organizations at the national, institutional, and international levels (106, 151, 152). Multimodal AI precisely and symbiotically improves adherence to evidence-based medicine in cancer palliative care for patients with genitourinary malignancies (106, 152, 153).

### 3.10 Explainability and human–AI collaboration in multimodal models for precision genitourinary malignancies

#### 3.10.1 The need for explainability

Standard metrics were used to evaluate the performance of several ML models like Random Forest (RF), Support Vector Machine (SVM), DNNs, Logistic Regression (LR), K-Nearest Neighbor (KNN), and Naive Bayes (NB). It has been elucidated that the results from DNNs (as one of those modern DL and multimodal fusion architectures) have shown more accuracy, recall, and precision in clinical decision-making, and genitourinary malignancies are no exception (154, 155).

#### 3.10.2 Approaches addressing explainability

To improve the transparency and explainability of multimodal AI and remove the opaque nature of CNNs (black boxes), integrating explainable AI (XAI) approaches like SHapley Additive exPlanations (SHAP), Local Interpretable Model-agnostic Explanations (LIME), and Partial Dependence Plots (PDPs) into multimodal AI has been shown to accelerate more precise and informed clinical decisions by uro-oncologists and uro-surgeons (154, 155).

In detail, it has been demonstrated that SHAP and LIME can tailor the quantification of the current contribution of clinical or genomic variables in patients with genitourinary malignancies. LIME has shown efficacy in tumoral and non-tumoral gene expression data for patients with bladder cancer, supporting the explainability of acquired clinical outcomes. Meanwhile, feature importance heatmaps, Grad-CAM, or saliency maps in imaging have introduced the localization of tumor lesions in MRI, driving the classification of patients with genitourinary malignancies. Moreover, attention mechanisms have highlighted which modality or feature mostly influences the model’s prediction (e.g., emphasizing MRI lesion size over PSA kinetics). All in all, these methods not only help clinicians verify whether AI-based decisions align with their currently established clinicopathological knowledge about their patients, but they can also decipher when models might be biased by spurious correlations (156–159).

### 3.11 The necessity for external validation of AI data in precision genitourinary malignancies

While many published studies report high performance on internal datasets, true clinical utility requires validation on an external dataset, ideally from multiple institutions with diverse patient populations and imaging protocols. In this manner, the ANN-TRUS system can be one of those shining examples in improving the detection and prediction of PCa, through external validation of the computerized analysis of TRUS of the prostate, as well as Gleason grading on RAPR specimens (160, 161).

To clinically interpret the data from lesion-specific histopathological EPE on MRI, currently-used multimodal algorithm-based AI were utilized for patients with localized, visible clinically significant PCa (or those with suspicious findings). Specifically, Gleason grading, pathology-based data, DWI, as well as ADC values were used at the patient and sextant levels. Then, to evaluate the sensitivity, specificity, and accuracy of the model’s diagnostic efficacy (in terms of lesion number and tumor volume), and the model’s predictive values (in terms of grading at the lesion-specific level), multicenter external data testing is highly recommended to improve diagnostic precision for these patients (162–164).

In another context, to clinically interpret data from currently-used algorithms like ANN-based ones in multimodal AI for patients with localized PCa, and to have a side-specific EPE risk assessment tool for those who have undergone RAPR, multi-institutional external validation is critically important. With multi-institutional external validation, increased accuracy, safety, and data generalizability can be achieved, as well as individualized nerve-sparing surgical procedures, and reduced treatment-related side effects (impotence and incontinence) during RAPR (165).

### 3.12 Human-centered design AI and clinician engagement in precision genitourinary malignancies

Successful adoption of AI in precision uro-oncology depends on designing tools that complement rather than disrupt clinical workflows. Early and sustained engagement with end users—urologists, surgeons, oncologists, radiologists, pathologists, and oncology nurses—ensures the capability of AI systems to clinically answer relevant questions, intuitively present information, accurately empower clinical decision support systems, and respect clinician autonomy. In other words, human–AI collaboration-based models have the potential to envision AI systems as decision-support tools that complement, rather than replace, clinical expertise in genitourinary malignancies (166, 167). As a prime example, multimodal AI could pre-populate tumor board dashboards with a comprehensive understanding of risk stratification outputs, while still allowing clinicians, uro-oncologists, and uro-surgeons to carefully review underlying imaging and pathology. Such hybrid workflows promise to preserve clinician agency and accountability, which is ethically and legally essential for the clinical management of patients with genitourinary malignancies.

To support the clinical interpretation of pre-operative MRI for patients with PCa, designing pilot studies and usability testing of XAI can identify barriers, such as alert fatigue or workflow misalignment, enable contextual perception of real-world practices, prioritize the clinical values and concerns of uro-oncologists, and carefully qualify clinical decisions before large-scale deployment (166, 168).

### 3.13 Limitations, regulatory, and ethical considerations of AI in precision genitourinary malignancies

As a transformative tool, multimodal AI systems utilized in clinical settings for precision genitourinary malignancies must comply with evolving regulatory frameworks, such as the European Medical Device Regulation (MDR), as well as U.S. FDA guidance for AI and ML-based software as a medical device (ML-based SaMD). These systems aim to customize and optimize treatment efficacy according to each patient’s genetic predisposition, while maintaining autonomy, avoiding algorithmic bias, and ensuring equitable access to AI-driven healthcare (169, 170). However, they still present limitations (L), and key regulatory (R) requirements include:

• L: Complex inter-modal dependencies in multimodal approaches, trial underrepresentation (certain racial, ethnic, or age groups), and disparities in treatment modalities. To mitigate these issues, fairness-aware training algorithms and subgroup analyses to identify differential performance are highly recommended. Developing visualization tools that jointly and prospectively represent imaging, molecular, and clinical drivers of AI decisions in a clinically-intuitive format for clinicians also seems logical (171).
• L: Demonstrating safety and efficacy through external clinical validation.
• R: Statistical harmonization strategies are needed in multicenter genomics and radiomics features extracted from prostate T2-weighted MRI (172). This highlights the importance of pre-validation to identify erroneous data before establishing a multimodal AI, and generating data repositories (173).
• R: Ensuring patient satisfaction and confidence in the use of such an integrative approach in a clinical setting (174).
• L: The integration of complex, high-dimensional data sources, such as imaging, histology, molecular pathology, genomics, and epigenomics, into a single predictive framework requires interdisciplinary knowledge to be understandable for a wide range of clinical specialties and bioinformaticians (171).
• R: Transparent documentation of algorithms is necessary and must be updated with authorized access.
• L: Concerns exist about inner biases or misunderstanding of the algorithms, or misimplementation of those algorithms in a daily clinical setting, leading to biased cancer palliative care (175).
• R: Establishing benchmark datasets and harmonization techniques, such as ComBat for radiomics aimed at standard preprocessing pipelines, is crucial to reduce the vast inter-institutional variability in imaging protocols, pathology digitization, and genomic platforms (achieving 70% accuracy and 78% Area Under the Curve (AUC)) (172).
• R: Generalizability of multimodal AI is essential and can only be achieved through validation in larger, more prospective multicenter datasets, as well as transparent reporting for patients with clinically-significant PCa, leading to transition from academic proof-of-concept to real clinical practice (176).
• R: Data safety, cybersecurity, familiarity, and interpretability are necessary for accreditation of clinical decisions for urologists, uro-surgeons, uro-oncologists, as well as patients (169, 174, 177).
• R: Post-market monitoring must be implemented to detect performance drift over time.
• R: Keeping pace with the swift increase and substantial progress in the development of AI-based technologies is essential (169).

R: Demanding precise and careful alignment between multimodal AI and real-world clinical workflows, regulatory requirements, and ethical principles, with the aim of integrating it into routine personalized urologic oncology as a translational roadmap.

### 3.14 Large language models in AI in precision genitourinary malignancies

In recent years, the rapid advancement of AI architectures has significantly broadened the functional scope of multimodal learning frameworks within biomedical research and clinical oncology. Among these technological developments, transformer-based DL models have emerged as a particularly influential paradigm, owing to their capacity to capture long-range dependencies within highly complex datasets (178–180). Initially introduced for natural language processing applications, transformer architectures utilize attention mechanisms, enabling models to selectively prioritize relevant features across heterogeneous data modalities. In the setting of PCa and other urologic malignancies, transformer-driven multimodal frameworks have been increasingly implemented to integrate diverse sources of information, including radiologic imaging, histopathological WSI, genomic sequencing datasets, and structured clinical variables. Through the utilization of cross-modal attention mechanisms, these models are capable of identifying associations between imaging phenotypes and underlying molecular signatures, thereby enhancing predictive performance for tumor aggressiveness, therapeutic response, and patient survival outcomes (178, 179, 181, 182).

Concurrently, the development of large language models (LLMs) has created new opportunities for incorporating unstructured biomedical knowledge into multimodal clinical decision-support systems. LLMs, which are trained on extensive corpora of biomedical and clinical textual data, possess the capability for contextual reasoning, information synthesis, and structured knowledge extraction from a wide array of textual sources, including electronic health records, radiology reports, pathology narratives, and the scientific literature (183–185). When integrated into multimodal AI pipelines, these models can serve as semantic interpreters that convert unstructured clinical narratives into machine-readable representations, thereby enabling their seamless integration with imaging, molecular, and demographic datasets. Such hybrid multimodal architectures have demonstrated considerable potential in facilitating clinical documentation analysis, automated report generation, and knowledge-guided diagnostic reasoning, ultimately assisting clinicians in navigating complex decision-making processes within the domain of precision uro-oncology.

Moreover, generative AI models, particularly generative adversarial networks (GANs) and diffusion-based architectures, have recently assumed an increasingly important role in improving the performance of multimodal AI systems in oncology research, including PCa imaging (186, 187). These generative frameworks, composed of two NNs (a generator and a discriminator), are capable of producing realistic medical images from synthetic data, augmenting limited datasets, and reconstructing missing data modalities. This is especially advantageous in clinical scenarios characterized by incomplete multimodal information, overcoming some limitations of DL-based approaches by providing image-to-image translation (186, 188). Within kidney and PCa research, generative AI approaches have been investigated for applications such as generating high-resolution histopathological images, segmentation (like an autonomous kidney segmentation technique, namely SegTGAN based on conventional GANs), enhancing MRI reconstruction, WSI resolution, or PSMA-PET imaging (through automated lesion detection and segmentation), increasing diagnostic precision, improving prediction (through dose prediction for intensity modulated radiation therapy), and simulating TME characteristics from partial datasets (186, 188–194). These functionalities not only contribute to improved robustness and generalizability of multimodal predictive models, but also promote data harmonization across different clinical institutions. Collectively, the convergence of transformer architectures, LLMs, and generative AI represents a significant advancement in multimodal AI, offering a more comprehensive representation of tumor biology and facilitating the progression toward clinically implementable precision oncology systems (187, 191).

### 3.15 Multi-omics-based models in AI in precision genitourinary malignancies

Recently, the systematic integration of data derived from the genome, epigenome, transcriptome, metabolome, microbiome, immunome, radiome, and proteome has been simplified through the emergence of “multi-omics technologies.” This has enabled fundamental progress in preclinical research via several bioinformatics platforms aimed at analyzing molecular subtypes, radiologic findings, histological evaluations, complex pathological mechanisms, integrating imaging, genomic or epigenomic pathways, and patient-specific characteristics, particularly in oncology (83, 195, 196).

Accordingly, there are many powerful biological tools for data analysis capable of integrating genomics, transcriptomics, and epigenomics data. Two prominent examples are iCluster/iClusterPlus, and Similarity Network Fusion (SNF), which use sophisticated statistical modeling implemented in the R language, and network-based methods, respectively. They have been considered for depicting distinct biological subgroups and identifying potential diagnostic and therapeutic biomarkers (83, 195).

Overall, multi-omics, AI, and ML can be considered powerful tools to underscore the metabolomic, genomic, epigenomic, histopathologic, immunopathophysiological, clinical, and radiological underpinnings of PCa. They facilitate the discovery of correlations among these multi-layered omics data, with the aim of early diagnosis, precise risk stratification, personalized treatment, and response monitoring, when compared to conventional single-omics analyses (83, 195, 197, 198).

In prostate cancer (PCa), integrating multi-omics data (e.g., metabolomics, radio-pathomics, and transcriptomics) with heterogeneous, high-dimensional data (non-linear correlations) acquired from CNNs, and analyzing them via ML and DL, can provide a more comprehensive understanding of its multifaceted nature. This integrative approach can lead to precise diagnosis/prognosis, biomarker discovery, differentiation between patients with PCa and BPH, effective dose-painting strategies in prostate radiation therapy (based on lumen and epithelium density for high- or low-grade PCa), risk stratification (progression from mHSPCa to mCRPCa), and recurrence prediction (199–204). Moreover, it can facilitate effective therapeutic procedures, elucidate cancer immunopathophysiology, predict the efficacy of immunotherapy (e.g., single-agent anti-PD-1/PD-L1 immunotherapy), reveal breakthroughs in intra-tumoral heterogeneity, and establish a cohesive research framework in cancer biology (83, 196, 199, 205–208).

For example, a comprehensive study integrating genomic data from single-cell DNA sequencing (scDNA-seq) or single-cell whole-genome sequencing (scWGS) and AI demonstrated critical insights into tumor mutational landscapes/evolutionary architectures, and genome methylation/expression patterns of RNA in patients with advanced PCa (carrying well-defined phenotypes) and papillary renal cell carcinoma, respectively (66).

In addition, in a study analyzing circulating tumor DNA (ctDNA) as a minimally invasive blood-based assay, the authors sought to detect mutation and copy-number alterations in patients with PCa. Genomic subtypes of mCRPCa tumors with well-defined phenotypes were classified by whole-transcriptome RNA-sequencing (RNA-seq) and IHC assays for protein expression. A Patient Derived Xenograft (PDX) model was then used to interrogate epigenetic nucleosome patterns in ctDNA, confirming the activity of key genomic regulators in differentiating androgen receptor prostate cancer (ARPCa) and neuroendocrine prostate cancer (NEPCa) phenotypes (209).

Interestingly, integrating bulk RNA sequencing, single-cell RNA sequencing (scRNA-seq), and spatial transcriptomics with IHC and immunofluorescence staining proved helpful in demonstrating the immune profile of patients with PCa, as well as monitoring treatment response. The results of a study highlighted intracellular signaling between FAP+ fibroblasts and SPP+ macrophages [tumor-associated macrophages (TAMs)], as well as notable tumor-specific intercellular signaling pathways, such as CSF1/CSF1R and CXCL/ACKR1, suggesting an immunosuppressive TME and a worsened prognosis (210).

In renal malignancies, given the various patterns of DNA methylation and the existing challenges in correlating DNA methylation with gene expression, TCGA multi-omics data can be a valuable method for demonstrating hypermethylation in the promoter and body of tumor suppressor genes, microRNAs, and gene clusters and families (cadherins, proto-cadherins, claudins, and collagens), as well as diversity in their immune functions. For example, altered expression of IL-17RE and immune checkpoint genes (HHLA2, SIRPA, and HAVCR2) correlated with hypermethylated patterns in patients with papillary renal cell carcinoma. Integrating data from hypermethylation and expression levels can profoundly improve machine learning models by using features extracted from single and multi-omics data to differentiate between early and late stages of tumor development in patients with papillary renal cell carcinoma, leading to patient stratification and the development of renal cancer precision medicine (211).

In bladder cancer, clinical application of multi-omics data has facilitated the determination of molecular subtypes of muscle-invasive bladder cancer (through convergent clustering by mRNA, long non-coding RNA (lncRNA), and miRNA expression), intra-tumor heterogeneity, transcriptional dynamicity (e.g., low *PPARG* expression, indicative of an aggressive phenotype), and stratified targeted therapy, achieved via computational integration of TCGA datasets (212–214). Integrative multi-omics analysis of muscle-invasive bladder cancer has revealed prognostic values for frontline chemotherapies or immunotherapies, particularly given the limited number of responders to cisplatin-based chemotherapy and PD-L1 blockade immunotherapy among these patients. Low expression of MTAP/CDKN2A/2B was associated with a poor response to anti-PD-L1 immunotherapy and a worsened prognosis (213, 215).

## 4 Future perspectives and discussion

Multimodal AI represents a fundamental methodological shift in urologic precision oncology by enabling computational fusion of heterogeneous, high-dimensional datasets through advanced machine learning and deep learning architectures. Unlike conventional statistical or rule-based systems, multimodal AI frameworks employ techniques such as convolutional neural networks, GNNs, transformer-based models, and ensemble learning to jointly analyze radiological imaging, digital histopathology, genomic and epigenomic profiles, molecular biomarkers, and structured clinical variables. This integrative modeling allows for the extraction of latent, cross-modality representations that more accurately reflect tumor biology and disease behavior (54, 56, 216).

In AI-enabled diagnosis and treatment planning, these frameworks move beyond descriptive risk stratification toward predictive and prescriptive modeling. By using architectures such as CNNs, GNNs, transformer-based models, and ensemble learning, multimodal AI can generate clinically actionable outputs, including probabilistic estimates of tumor aggressiveness, extracapsular extension, lymph node involvement, biochemical recurrence, prediction of response to neoadjuvant chemotherapy, and treatment resistance. Such outputs support individualized surgical planning, radiotherapy adaptation, and systemic therapy selection (44, 56, 75).

The translational strength of multimodal AI lies in its ability to model non-linear interactions and longitudinal disease dynamics by integrating serial imaging, biomarker kinetics, and treatment timelines. The incorporation of explainable AI techniques, including attention mechanisms and feature attribution, further enhances interpretability and facilitates integration into multidisciplinary clinical workflows, especially in predicting post-surgical complications and tumor grading in patients with primary PCa (60, 61).

Future advancements will rely on integrating emerging data modalities, such as liquid biopsy-derived circulating tumor DNA, spatial and single-cell transcriptomics, immune profiling, and patient-generated health data, into unified AI architectures. These developments are expected to improve modeling of intratumoral heterogeneity, clonal evolution, and therapy-induced selective pressures (65, 217).

Nonetheless, successful clinical translation requires addressing AI-specific challenges, including data harmonization, external validation, model generalizability, regulatory oversight, and ethical governance. Through interdisciplinary collaboration among clinical specialists, continued methodological refinement, and collaborative implementation, multimodal AI has the potential to align algorithmic development with real-world clinical practice, operationalize precision medicine in urologic oncology, and bridge computational innovation with tangible patient benefit (216).

## 5 Conclusion

Multimodal AI represents a paradigm shift in urologic precision oncology, offering a powerful tool to bridge the gap between complex biological data and individualized patient management. By integrating imaging, histopathology, genomic, epigenomic, molecular, and clinical information, these models can enhance diagnostic accuracy, prognostication, and therapeutic planning. The future of multimodal AI in urologic precision oncology lies in the integration of increasingly diverse data streams, including liquid biopsies, spatial transcriptomics, and patient-generated health data—into more adaptive and comprehensive models. Successful clinical translation requires careful attention to data integration, interpretability, workflow implementation, and ethical standards. With continued methodological advancement and collaborative efforts, multimodal AI is poised to transform urologic oncology, advancing the principles of precision medicine from computational innovation to real-world patient impact.

Accordingly, to translate these computational innovations into clinically meaningful interventions for patients with genitourinary malignancies, further investigations and interdisciplinary or cross-disciplinary collaboration among oncologists, radiologists, interventional radiologists, radiation oncologists, nephrologists, urologists, uro-oncologists, interventional uro-surgeons, onco-surgeons, uro-surgeons, clinical andrologists, clinical pathologists, internal medicine specialists, endocrinologists, bioinformaticians, data scientists, AI specialists, computer engineers, software engineers, biomedical engineers, computational biologists, palliative care specialists, neurologists, clinical biochemists, clinical immunologists, clinical geneticists, cellular and molecular cancer immunotherapists, clinical physiologists, cellular and molecular biologists, molecular immunobiologists, pharmacologists, personalized medicine specialists, molecular medicine specialists, translational medicine specialists, translational andrology and urology specialists, evidence-based medicine specialists, experimental medicine specialists, molecular pathologists, computational pathologists, molecular biotechnologists, medical biotechnologists, cancer biology researchers, medical laboratory scientists, basic medical scientists, biomolecule scientists, disease-specific molecular biomarker specialists, and health system coordinators are highly recommended.
